# Does a low‐carb, high‐fat diet change muscle insulin receptor concentration and body composition in a swine biomedical model?

**DOI:** 10.14814/phy2.71124

**Published:** 2026-09-29

**Authors:** Natalie Acosta‐Castellanos, Shelly Tiede, Christopher J. Byrd, Pawel Borowicz, Jennifer M. Young, Eric P. Berg

**Affiliations:** ^1^ Department of Animal Sciences North Dakota State University Fargo North Dakota USA; ^2^ Ralco Agriculture Marshall Minnesota USA

**Keywords:** diet, insulin receptor, ketogenic, swine

## Abstract

Our objective was to evaluate body composition and muscle insulin receptor concentration resulting from consumption of a low‐carbohydrate, high‐fat diet using swine as a surrogate for humans. Two treatments, a control standard swine feed (CON; *n* = 7) and test diet where beef tallow replaced the corn carbohydrate (LCHF; *n* = 8), were fed for 32 days to post‐pubertal gilts (Landrace × Yorkshire). Body weight (BW), subcutaneous fat (FD), daily gain (ADG), daily feed intake (ADFI), and fasting blood chemistry were measured. Organ weights, and pancreas, longissimus, and semimembranosus samples were collected from three gilts per treatment. LCHF gilts had reduced ADG, ADFI, FD, and visceral fat (*p* = 0.09, 0.04, 0.06, and 0.03; respectively) with similar BW (*p* = 0.18). Blood chemistry parameters were not affected but for fasting glucose, which tended to be higher for LCHF (*p* = 0.054). The percentage (*p* = 0.09) and number (*p* = 0.07) of active pancreatic β‐cells tended to be higher for LCHF gilts, as was the concentration of semimembranosus insulin receptors (*p* = 0.04). Short‐term consumption of a low‐carb, high‐fat diet improved body composition, insulin production mechanics, and muscle insulin receptor density in a swine biomedical model.

## 1 INTRODUCTION

Dietary carbohydrates and their impact on the production and release of insulin have been implicated as having a causal role in the pathological accumulation of body fat (Hall et al., 2016). For this reason, one of the most popular diets among healthy and active adults is the very low carbohydrate (low‐carb), high‐fat, ketogenic diet. Consumption of carbohydrates on a standard ketogenic diet is very low (<50 g per day or 10% of daily energy intake) and fat intake is high (>75% of total energy); therefore, the typical ketogenic base diet is comprised of meat, poultry, fish, eggs, nuts, seeds, and oils and avoids grains, most fruits, starchy vegetables, and sugary foods (Wong et al., 2021).

The therapeutic goal of the low‐carb, high‐fat dietary strategy is to induce nutritional ketosis to simulate a physiological state similar to fasting. Thus, the ketogenic diet works on lipid and glycemic regulation through the dietary maintenance of a negative energy balance, resulting in reduced insulin secretion and fewer insulin release fluctuations. Lower circulating glucose and insulin shift metabolism from a state of energy storage to a state of fat catabolism, resulting in weight loss via body fat reduction (Al‐Khalifa et al., 2009; Harvey et al., 2018). Despite their popularity, the use of ketogenic diets as a therapeutic tool has not received widespread endorsement from clinicians due to possible side effects from prolonged applications such as fatigue, headache, hypoglycemic episodes, constipation, and a rise in serum LDL‐cholesterol and triglycerides (Paoli, 2014; Wong et al., 2021).

Humans are not reliable participants in diet‐related studies. Their participation and adherence to the research protocol are critical to the quality of the research data. Assessing the impact of food and food combinations on physiological and metabolic changes can be more effectively controlled in animal studies. Therefore, using swine as a biomedical model for humans, this study aimed to investigate changes in muscle insulin receptor concentration and body composition when carbohydrate energy is replaced with beef tallow as the main energy source in a nutritionally balanced swine diet.

## 2 METHODS

All procedures were approved by the North Dakota State University (NDSU) Institutional Animal Care and Use Committee (Protocol #20220002).

### 2.1 Animals and dietary treatments

Fifteen post‐pubertal, non‐pregnant, non‐lactating gilts (Landrace × Yorkshire) with an initial average body weight (BW) of 170 ± 18 kg and an initial average age of 236 ± 3 d were individually penned (1.22 × 2.44 m) and fed at NDSU's Animal Nutrition and Physiology Center. A conventional swine diet was provided during the adaptation period (7 d) prior to the experiment. Gilts were paired by weight and then randomly assigned within the pair to one of two experimental diets. Dietary treatments followed the recommended dietary guidelines from the National Research Council (Weiss & Tebbe, 2019) dietary requirements for swine of this age, stage of reproduction, and sex. The control diet (CON; *n* = 7) was a complete, nutrient‐balanced maintenance ration (National Research Council (NRC), 2012), while the low‐carbohydrate, high‐fat diet (LCHF; *n* = 8) replaced corn in the CON diet with beef tallow as the energy source (Table 1). Soy hulls were added to the LCHF diet as a non‐nutritive ingredient to match the dietary consistency of the CON. The CON and LCHF dry ingredients were prepared at the Northern Crops Institute feed mill (Fargo, ND, USA) and beef tallow was added to the LCHF at the experimental facility. All gilts were provided 24‐h ad libitum access to water via standard nipple waterers. The dietary rations were fed daily at 0800 h at an average of 1.75% of each gilt's BW for 32 d. Measures of urine ketosis were taken using commercial ketone test strips (TRUEplus, catalog #56151‐0601‐50) in weeks three and four of the trial. Body weight and subcutaneous fat depth (FD; adjacent the 10th thoracic vertebra) were measured on d 0, 19, and 32 of the experiment. Subcutaneous fat depth was measured using an ALOKA SSD‐500 V ultrasound device (Hitachi Healthcare, Twinsburg, Ohio, USA). Feed offered and unconsumed weigh backs were recorded daily from d 13 to 31. Prior to d 13, weigh‐back of unconsumed feed was not recorded; therefore, those data are not presented. Average daily gain (ADG) from d 0 to 32 and average daily feed intake (ADFI) were calculated from d 19 to 31.

**TABLE 1:** Composition of the experimental diets presented as percentage of total dry matter basis.

Ingredients (%)	Treatments	
	CON	LCHF
Corn	85.82	0.00
Soybean meal	11.91	13.17
Limestone	0.83	0.15
Salt	0.45	0.45
Monocal 21P 18Ca	0.32	0.44
L‐Lysine	0.30	0.14
5202 EnMax G/F Mill 5.0 T	0.25	0.25
L‐Threonine	0.09	0.09
DL‐Methionine	0.04	0.18
Soy Hulls	0.00	64.41
Fat, beef tallow	0.00	20.75

Abbreviations: CON = complete, nutrient balanced ration (control), LCHF = beef tallow replaced corn as the main energy source.

### 2.2 Tissue collection

Blood was collected via jugular venipuncture after a 16 h fast on d 0, 18, and 32. Blood chemistry, gas, and hematology panels were immediately determined using the i‐STAT cartridge‐based analyzer (CG8+ cartridge; Abbot Point of Care, Kansas City, Missouri, USA) for Na, K, iCa (ionic calcium), glucose, hematocrit, hemoglobin, pH, partial pressure of CO_2_, partial pressure of O_2_, total CO_2_, bicarbonate, base excess, and oxygen saturation.

After weighing on d 32, three median‐weight gilts from each treatment were selected for euthanasia. Gilts were euthanized on d 33 under United States Department of Agriculture Food Safety and Inspection Service (USDA FSIS) supervision following USDA‐FSIS humane and sanitary standards. Euthanasia was conducted using head‐to‐heart electrical stunning, followed by exsanguination. Eviscerated body weight (EBW) and liver, kidney, visceral fat, pancreas, and heart weights were recorded and expressed as a percentage of final BW. Samples of kidney, pancreas, *longissimus thoracis* (adjacent the 10th thoracic vertebra), and *semimembranosus* were collected and placed in 10% neutral buffered formalin for immunostaining. Additional 50 g samples were collected from liver and longissimus, packed in Whirl‐Pak® bags, and stored at −20°C for proximate analysis. After tissue collection, samples in formalin were transferred to 70% ethanol for further processing.

### 2.3 Proximate analysis

Each time a CON or LCHF diet was manufactured, samples were collected. At the end of the experiment, samples were combined within their respective treatments, mixed, subsampled, and analyzed in duplicate for nutrient composition. Dietary analysis was conducted according to the official methods of AOAC International (Latimer Jr., 2023). Analysis of dry matter (DM; AOAC 934.01), ash (inorganic component; AOAC 942.05), calcium (AOAC 965.17), crude fat (CF; AOAC 968.08), phosphorus (AOAC 920.39), crude protein (AOAC 984.13), and acid detergent fiber (cellulose and lignin; AOAC 973.18: A‐D) were determined for both diets (Latimer Jr., 2023). Neutral detergent fiber (NDF; cellulose, hemicellulose, and lignin) was determined from the procedural method of Mertens (2002). Total non‐fiber carbohydrate (NFC) was calculated utilizing the following equations (Weiss & Tebbe, 2019):

$$\mathrm{NFC}=100-\left[\mathrm{ash}\;\left(\%\right)-\mathrm{CP}\;\left(\%\right)-\mathrm{NDF}\;\left(\%\right)-\mathrm{CF}\;\left(\%\right)\right]$$

and gross energy (Kcal/kg of feed) was calculated as (Weiss & Tebbe, 2019)

$$\begin{array}{c} \text{Kcals}/\mathrm{kg}\;\text{of feed}\;\left(\text{gross energy}\right)=\left[\mathrm{CP}\;\left(\%\right)\times 56\right]+\left[\mathrm{NFC}\;\left(\%\right)\times 42\right]\\ \;+\left[\mathrm{CF}\;\left(g\right)\times 94\right] \end{array}$$

*Longissimus thoracis* and liver samples were freeze‐dried and ground prior to proximate analysis of dry matter, insoluble ash, crude fat, P, and Ca.

### 2.4 Immunofluorescent staining

Formalin‐fixed samples from *longissimus*, *semimembranosus*, kidney, and pancreas were processed using the Leica ASP300S fully enclosed tissue processor (Leica Biosystems, Deer Park, IL) and embedded in paraffin (Tissue Tek TEC5; Sakura Finetek Inc., Torrance, CA). To expose the cross‐section of the tissue, paraffin blocks were sectioned in 5 μm intervals on a Leica RM2255 semi‐automatic rotary microtome (Leica Biosystems, Deer Park, IL) and mounted on glass slides. Before staining, paraffin was removed from slides with three xylene washes for 30 s each followed by application of descending concentrations of ethanol to remove xylene residues from tissue. Finally, distilled water was used to rehydrate tissues prior to heat induced antigen recovery. Sodium citrate buffer of 10 mM at pH 6 and a Retriever 2100 (Electron Microscopy Sciences, Hatfield, PA) set at 100°C for 30 min were used to expose target proteins. Tissues were then blocked with 5% Normal Goat Serum for 1 h (S‐1000‐20; Vector Laboratories, Inc., Newark, CA).

Muscle and kidney samples were stained to detect insulin receptor presence and density using mouse anti‐insulin receptor alpha as the primary antibody (ab136650; Abcam Laboratories, Cambridge, UK) diluted 1:100 in 1% bovine serum albumin (10791‐790; VWR International, Radnor, PA) for 1 h at room temperature (21°C). Pancreas slides were stained to detect insulin content using mouse anti‐insulin antibody (ab46707; Abcam Laboratories, Cambridge, UK), diluted 1:1000 in 1% BSA for 1 h at room temperature (21°C). The secondary antibody, goat anti‐mouse IgG (CF633, 20120; Biotium, Fremont, CA), was diluted 1:250 in 1% bovine serum albumin, applied to all the slides, and incubated at room temperature for 1 h. Slides were treated with DAPI as a nuclear counterstain for 5 min (40043; Biotium, Fremont, CA). Finally, the slides were mounted with the EverBrite Hardset mounting medium (23,003; Biotium, Fremont, CA) to preserve the fluorescent dyes.

### 2.5 Image analysis

Slides from the mid cross‐section of *longissimus*, *semimembranosus*, kidney, and pancreas were analyzed with ZEN Imaging software (Carl Zeiss AG., Oberkochen, Germany). Three images were gathered of each slide (one slide per tissue per animal) at 200× magnification using the Zeiss Axio Observer Z1 inverted confocal microscope. The ZEN software was programmed to use two different lasers simultaneously. The laser 405 was set for DAPI staining to detect a blue fluorescence, while laser 639 was set for insulin or insulin receptor staining to detect far‐red fluorescence. Images were converted from the Laser Scanning Microscope format to Tagged Image File Format using Carl Zeiss AxioVision Rel. 4.8.2 Software (Carl Zeiss AG, Oberkochen, Germany). Insulin image analysis, as an indicator of pancreatic β‐cell activity, was performed using the Image‐Pro software (Media Cybernetics, Inc., Rockville, MD) to identify the number of cells, cell area in the islet, number of active β‐cells, β‐cell area within each islet, number of islets/area of tissue, and islets area versus total area of the image based on size (area covered in pixels) and intensity of fluorescence. The insulin receptor density in the kidney, *longissimus*, and *semimembranosus* was determined with ZEN Imaging software (Carl Zeiss AG., Oberkochen, Germany) using the relative intensity as the expression of the fluorescent dye on a specific area of the cross‐section.

### 2.6 Statistical analysis

All data were analyzed using the MIXED procedure of SAS (v. 9.4, SAS Inst. Inc., Cary, NC). For BW, FD, and blood chemistry parameters, a repeated measures design was utilized, fitting diet, day, and diet × day as fixed effects and animal repeated across time as the repeated measure. Average daily BW gain was analyzed as a single trait across the entire test period (d 0–32) and ADFI from d 13 to 31 fitting diet, time, and diet × time interaction as fixed effects and animal repeated across time as the repeated measure. For EBW and organ traits, a model was fit including diet as a fixed effect. For all models including a repeated measures statement, different covariance structures were tested and the best fit based on AICc (Akaike Information Criterion corrected) was chosen. Least‐squares means were calculated to determine differences between dietary treatments and were considered significant at *p* ≤ 0.05 and tendency identified as 0.05 < *p* ≤ 0.10.

## 3 RESULTS

### 3.1 Proximate analysis

As designed, the LCHF diet contained more crude fat and less non‐fiber carbohydrates than CON (Table 2). Diet formulations were estimated to contain 4000 kcals/kg of diet. Actual diet analysis had the CON providing 3993 kcals and LCHF at 3448 kcals/kg of feed.

**TABLE 2:** Proximate analysis^a^ of the experimental diets: A complete, nutrient‐balanced maintenance ration (control, CON) and a low‐carbohydrate, high‐fat diet that replaced corn with beef tallow as the main energy source (LCHF).

Nutrient	Treatment	
	CON	LCHF
Dry matter (DM), % as fed	88.55 ± 0.06	91.10 ± 0.21
Ash, % DM	3.99 ± 0.05	5.48 ± 0.04
Crude protein (CP), % DM	13.84 ± 0.13	12.13 ± 0.00
Crude fat (CF), % DM	3.34 ± 0.01	22.48 ± 0.14
Calcium, % DM	0.520 ± 0.008	0.637 ± 0.029
Phosphorous, % DM	0.312 ± 0.0003	0.256 ± 0.004
Neutral detergent fiber (NDF), % DM	9.69 ± 0.07	44.31 ± 0.08
Acid detergent fiber (ADF), % DM	3.05 ± 0.06	32.13 ± 0.45
Total non‐fiber carbohydrates (NFC)^c^, % DM	69.14	15.6
Calculated gross energy^b^, Kcal/kg	3993	3448

^a^ Results presented as the mean ± standard deviation of the two duplicates for each sample.

^b^ Total NFC = 100 − ash (%) − CP (%) − NDF (%) − CF (%).

^c^ Calculated gross energy (Kcal/kg) = CP (%) × 56 + NFC (%) × 42 + CF (g) × 94.

### 3.2 Performance characteristics

Performance characteristics are reported in Table 3. Average daily BW gain did not differ across treatment nor did treatments become different over time on test (*p* > 0.05). The ADFI calculated for days 19–32 differed (*p* = 0.02) by treatment with CON consuming more feed per day. Likewise, calculated kcals consumed per day showed that CON consumed more (*p* = 0.0006) calories of feed per day from Day 19 to 32 on test. However, the kcals consumed multiplied by the average daily weight gain (or loss) revealed a tendency toward a negative energy balance for LCHF versus CON (*p* = 0.08). Control gilts gained BW from d 0 to 19 (*p* = 0.009) but not from d 19 to d 32 (*p* = 0.52), resulting in an overall increase from d 0 to d 32 (*p* = 0.01) while LCHF gilts did not fluctuate and consistently maintained their BW (*p* > 0.59). Gilts in the LCHF started on test (d 0) with greater subcutaneous FD. Within treatment from d 0 to d 32, LCHF gilts had a 17.7% reduction in FD (*p* = 0.03) while the CON gilts had an increase of 14.0% (*p* = 0.20). Fat depth between LCHF and CON gilts did not differ (*p* = 0.36) at d 32.

**TABLE 3:** Least square means ± SE for performance characteristics of gilts consuming a complete, nutrient‐balanced maintenance ration (control, CON) and a low‐carbohydrate, high‐fat diet that replaced corn with beef tallow as the main energy source (LCHF) for 32 d.

Trait	Day	CON	LCHF	*p*‐value		
				Diet	Time	Diet × time
Body weight, kg	0	171.4 ± 5.57^a^	167.9 ± 5.21^ab^	0.16	0.06	0.17
	19	181.4 ± 5.57^b^	169.7 ± 5.21^ab^			
	32	183.7 ± 5.57^b^	168.2 ± 5.34^ab^			
Back fat depth, cm	0	2.29 ± 0.21^a^	2.99 ± 0.20^b^	0.07	0.78	0.06
	19	2.52 ± 0.07^a^	2.65 ± 0.07^ab^			
	32	2.61 ± 0.11^ab^	2.46 ± 0.11^a^			
ADG*, kg/d	0–19	0.53 ± 0.89	0.09 ± 0.83	0.19	0.23	0.41
	19–32	0.17 ± 0.89	−1.69 ± 0.83			
	0–32	0.38 ± 0.52	−0.63 ± 0.48	0.18		
ADFI*, kg/d	19–32	3.09 ± 0.19^a^	2.36 ± 0.18^b^	0.02		
Kcals × ADFI, kcals/d	19–32	12,342 ± 678^a^	8150 ± 634^b^	<0.01		
Kcals × ADG, kcals/kg BW gain	19–32	4875 ± 2883	−2693 ± 2697	0.08		
Body weight change*, %	0–19	5.68 ± 2.42^a^	1.32 ± 2.26^ab^	0.098	0.11	0.58
	19–32	1.34 ± 1.24^ab^	−0.86 ± 1.26^b^			
	0–32	7.02 ± 2.76	0.44 ± 2.76	0.12		

*Note*: ^ab^Means with different superscripts within a trait differ by *p* < 0.05.

* Traits analyzed as a repeated measure for d 0–19 and 19–32 and as a single trait for d 0–32.

### 3.3 Body composition

Final BW, EBW, and organ weights are reported in Table 4. Control gilts had smaller livers by weight (*p* = 0.047) and expressed as a percentage of final BW (*p* = 0.03). The CON gilts also had more visceral fat by weight (*p* = 0.04) and by percentage BW (*p* = 0.03). Final BW, EBW, and kidney, pancreas, and heart weights (actual and percentage of final BW) did not differ (*p* > 0.20) across dietary treatment.

**TABLE 4:** Least square means ± SE for final body weight, eviscerated body weight (EBW), EBW as percentage of final weight, organ weights, and organ weights as percentage of final weight collected on d 33 from gilts consuming a complete, nutrient‐balanced maintenance ration (control, CON) and a low‐carbohydrate, high‐fat diet that replaced corn with beef tallow as the main energy source (LCHF) for a period of 32 d.

Trait	CON	LCHF	*p*‐value
Final BW, kg	181.0 ± 6.32	172.2 ± 6.32	0.38
EBW, kg	118.8 ± 4.29	112.2 ± 4.29	0.34
EBW, %	66.65 ± 1.17	65.19 ± 1.17	0.79
Liver, g	1880 ± 71^a^	2166 ± 71^b^	0.047
Liver, %	1.04 ± 0.04^a^	1.26 ± 0.04^b^	0.03
Kidney, g	508.7 ± 41.6	468.0 ± 41.6	0.53
Kidney, %	0.28 ± 0.02	0.27 ± 0.02	0.80
Visceral fat, g	2660 ± 285^a^	1440 ± 285^b^	0.04
Visceral fat, %	1.46 ± 0.13^a^	0.84 ± 0.13^b^	0.03
Pancreas, g	187.3 ± 17.8	214.7 ± 17.8	0.34
Pancreas, %	0.10 ± 0.01	0.12 ± 0.01	0.20
Heart, g	538.0 ± 17.1	534.7 ± 17.1	0.90
Heart, %	0.30 ± 0.01	0.31 ± 0.01	0.39

*Note*: ^ab^Means with different superscripts within a row differ by *p* < 0.05.

### 3.4 Blood chemistry

Fasted blood chemistry, gas, and hematology traits are reported in Table 5. Differences across diet were observed for K concentration (*p* = 0.01) with higher concentrations of K in the LCHF group on d 18 (*p* = 0.02); d 0 and d 32 did not differ (*p* = 0.10 and 0.95, respectively). Furthermore, iCa concentration tended (*p* = 0.08) to be lower in CON compared to LCHF. Blood glucose concentration increased (*p* = 0.04) over time (d 0 to d 32) for gilts consuming the LCHF diet, while CON gilts did not differ in blood glucose levels on d 32 compared to d 0 (*p* = 0.32). There were no treatment differences for blood glucose concentrations on d 0 (*p* = 0.37), but by d 32, LCHF gilts had higher blood glucose levels than CON (*p* = 0.02). For hematocrit and hemoglobin, CON gilts had lower values on d 32 than on d 0 (*p* = 0.005), while LCHF gilts only tended to have a lower value on d 32 versus d 0 (*p* = 0.08).

**TABLE 5:** Least square means ± SE for blood chemistry parameters collected after fasting for 16 h from gilts consuming a complete, nutrient‐balanced maintenance ration (control, CON) and a low‐carbohydrate, high‐fat diet that replaced corn with beef tallow as the main energy source (LCHF) for a period of 32 d.

Trait	Day	CON	LCHF	*p*‐value		
				Diet	Time	Diet × time
pH	0	7.45 ± 0.03^ab^	7.41 ± 0.02^a^	0.38	0.13	0.14
	18	7.43 ± 0.02^a^	7.46 ± 0.02^ab^			
	32	7.54 ± 0.04^b^	7.47 ± 0.04^ab^			
Na, mmol/L	0	144.6 ± 0.88^a^	144.4 ± 0.82^a^	0.60	0.02	0.96
	18	142.1 ± 0.88^ab^	141.9 ± 0.82^b^			
	32	144.0 ± 0.88^ab^	143.4 ± 0.82^ab^			
K, mmol/L	0	5.17 ± 0.27^a^	4.53 ± 0.25^abcd^	0.01	0.02	0.22
	18	4.41 ± 0.13^c^	3.95 ± 0.12^d^			
	32	4.50 ± 0.15^bc^	4.49 ± 0.14^b^			
iCa, mmol/L	0	1.24 ± 0.08	1.26 ± 0.08	0.08	0.71	0.52
	18	1.17 ± 0.08	1.32 ± 0.08			
	32	1.09 ± 0.08	1.29 ± 0.08			
Glucose, mg/dL	0	88.4 ± 4.3^ab^	83.0 ± 4.0^a^	0.66	0.67	0.07
	18	88.0 ± 2.7^ab^	88.9 ± 2.5^ab^			
	32	83.9 ± 2.5^a^	92.5 ± 2.3^b^			
PCO_2_, mmHg	0	47.7 ± 3.8^ab^	52.9 ± 3.6^b^	0.39	0.21	0.56
	18	49.77 ± 3.8^ab^	47.73 ± 3.6^ab^			
	32	41.53 ± 3.8^a^	46.21 ± 3.6^ab^			
PO_2_, mmHG	0	36.6 ± 5.1	32.6 ± 4.8	0.71	0.37	0.83
	18	41.3 ± 8.8	37.3 ± 8.2			
	32	28.1 ± 3.0	29.8 ± 2.8			
Base Excess, mmol/L	0	8.29 ± 1.05	8.25 ± 0.98	0.72	0.21	0.73
	18	8.57 ± 1.05	8.75 ± 0.98			
	32	10.57 ± 1.05	9.38 ± 0.98			
HCO_3_, mmol/L	0	32.4 ± 1.1	32.9 ± 1.0	0.82	0.92	0.96
	18	32.7 ± 1.1	32.8 ± 1.0			
	32	33.1 ± 1.1	33.1 ± 1.0			
TCO_2_, mmol/L	0	33.9 ± 1.2	34.6 ± 1.1	0.78	0.96	0.94
	18	34.1 ± 1.2	34.1 ± 1.1			
	32	34.4 ± 1.2	34.5 ± 1.1			
sO_2_, %	0	63.4 ± 6.8	56.9 ± 6.4	0.63	0.59	0.83
	18	65.6 ± 6.8	62.6 ± 6.4			
	32	56.4 ± 6.8	58.0 ± 6.4			
Hematocrit, % PCV	0	41.9 ± 1.7^a^	41.3 ± 1.6^ab^	0.72	0.006	0.22
	18	40.4 ± 1.7^ab^	37.0 ± 1.6^bc^			
	32	34.6 ± 1.7^c^	37.1 ± 1.6^abc^			
Hemoglobin, g/dL	0	14.2 ± 0.6^a^	14.0 ± 0.5^ab^	0.71	0.007	0.21
	18	13.8 ± 0.6^ab^	12.6 ± 0.5^bc^			
	32	11.8 ± 0.6^c^	12.6 ± 0.5^abc^			

*Note*: ^ab^Means with different superscripts within a trait differ by *p* < 0.05.

### 3.5 Liver and *longissimus* fat content

Liver and *longissimus* samples were not impacted by diet for percentage of DM, ash/inorganic compounds, crude fat, phosphorus, or calcium in either tissue (Table 6).

**TABLE 6:** Least square means ± SE for proximate analysis of swine liver and longissimus thoracis collected on d 33 from gilts consuming a complete, nutrient‐balanced maintenance ration (control, CON) and a low‐carbohydrate, high‐fat diet that replaced corn with beef tallow as the main energy source (LCHF) for a period of 32 d.

Tissue	Trait	CON	LCHF	*p*‐value
Liver	Dry matter (DM), %	27.0 ± 1.2	28.3 ± 1.2	0.48
	Ash, % DM	5.10 ± 0.14	4.92 ± 0.14	0.41
	Crude fat, % DM	9.79 ± 0.41	9.47 ± 0.41	0.61
	Phosphorous, % DM	0.882 ± 0.088	0.789 ± 0.088	0.50
	Calcium, % DM	0.0174 ± 0.0014	0.0138 ± 0.0014	0.16
*Longissimus thoracis*	Dry matter (DM), %	32.9 ± 1.1	32.5 ± 1.1	0.84
	Ash, % DM	3.15 ± 0.18	3.22 ± 0.18	0.81
	Crude fat, % DM	27.6 ± 3.3	25.9 ± 3.3	0.73
	Phosphorous, % DM	0.546 ± 0.044	0.572 ± 0.044	0.69
	Calcium, % DM	0.0105 ± 0.0005	0.0111 ± 0.0005	0.39

### 3.6 Insulin and insulin receptor

Image analysis of pancreatic β‐cell activity and insulin receptor density are presented in Table 7. The percentage of active β‐cells was greater (*p* < 0.0001) in the LCHF gilts. Additionally, the percentage of the area occupied by β‐cells within the pancreatic islets tended to be greater (*p* = 0.06) for LCHF versus CON. The percentage of active β‐cells (Figure 1a,b) present in the LCHF pancreas was 2.81 times greater than CON, and the area occupied by β‐cells within the islets was 1.81 times larger than CON. The relative intensity of insulin receptors was significantly greater (*p* = 0.04) in the *semimembranosus* muscle from LCHF gilts compared to CON (Figure 1c,d). Diet did not significantly impact kidney or *longissimus* insulin receptor density (not pictured).

**TABLE 7:** Least square means ± SE for pancreatic β‐cell activity and density and relative fluorescence of insulin receptors present in kidney, *longissimus thoracis*, and *semimembranosus* collected on d 33 from gilts consuming a complete, nutrient‐balanced maintenance ration (control, CON) and a low‐carbohydrate, high‐fat diet that replaced corn with beef tallow as the main energy source (LCHF) for a period of 32 d.

	CON	LCHF	*p*‐value
*Activity of pancreatic β‐cells*			
Active β‐cells, %	42.4 ± 11.8^a^	90.7 ± 11.8^b^	0.04
β‐cells area, % of area within islets	30.8 ± 8.1	47.6 ± 8.1	0.16
Islet area, % of pancreas area	1.04 ± 0.12	2.26 ± 0.12	0.002
*Relative intensity of insulin receptors in different tissue types*			
Kidney	11.34 ± 1.03	8.77 ± 1.03	0.15
*Longissimus thoracis*	23.3 ± 3.2	28.3 ± 3.2	0.33
*Semimembranosus*	21.7 ± 2.6^a^	32.8 ± 2.6^b^	0.04

*Note*: ^ab^Means with different superscripts within a trait differ by *p* < 0.05.

**FIGURE 1:**
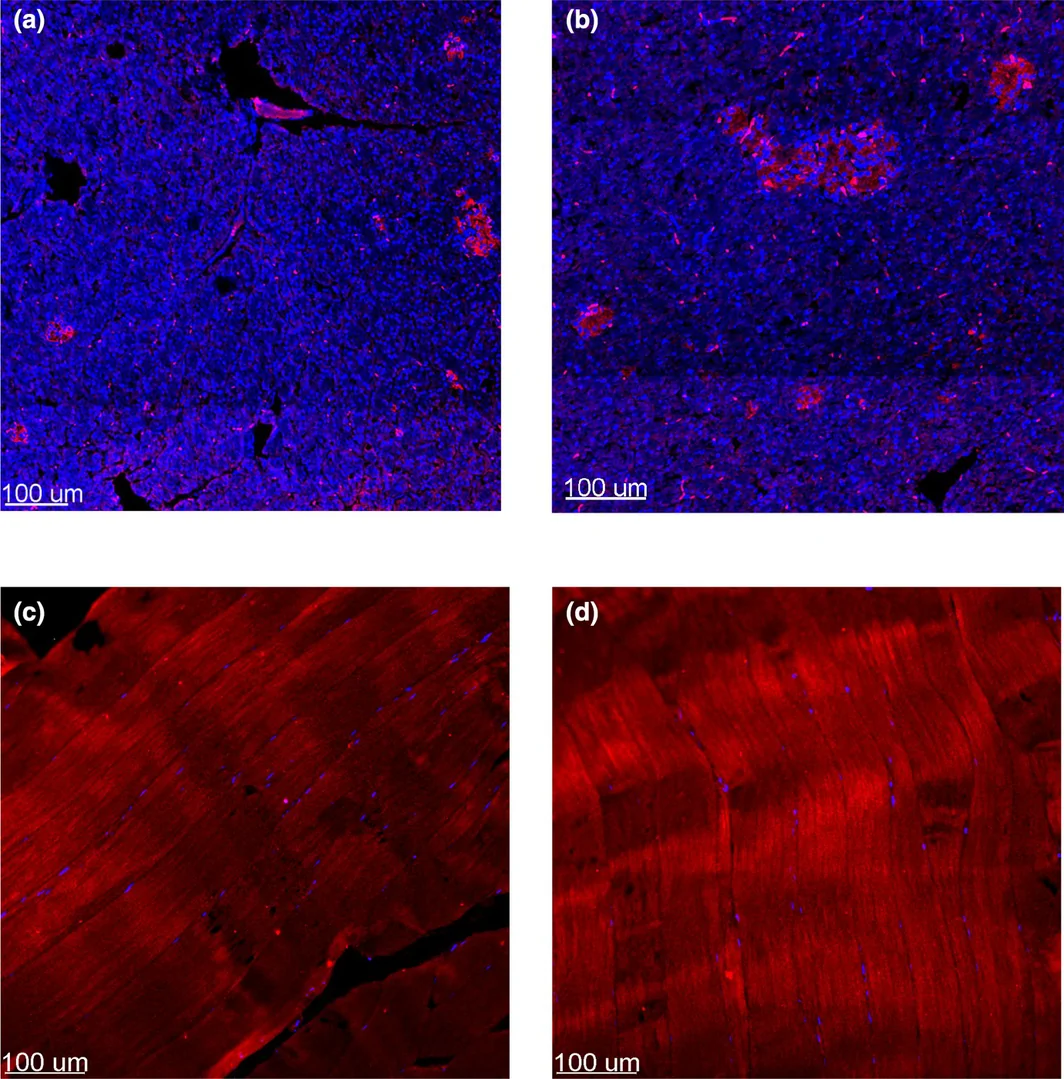
Pancreas tissue of mature gilts fed (a) a control diet (CON) and (b) a low‐carbohydrate, high‐fat diet, replacing corn with beef tallow (LCHF) as the main energy source; pancreatic sections were stained for insulin (red) using ab46707 at 1/1000 dilution. Muscle fiber sections cut longitudinally from swine *semimembranosus* muscle from mature gilts fed (c) a CON diet and (d) a LCHF diet; *semimembranosus* muscles were stained for insulin receptors (red) using ab36550 at 1/100 dilution. Blue dye represents DAPI‐stained nuclei for both tissues.

## 4 DISCUSSION

Given the limitation on the number of animals able to be used for this study, we decided to increase statistical power by limiting the animals used to one sex. Intact males at this age were not available, and castrated males are not relevant to human medicine; therefore, we utilized mature gilts for this study. We do understand that this limits the results to generalizations to females and not males. We also acknowledge the limitation of ex vivo analyses performed with only three biological replicates per group. This potentially limits the ability to detect differences between treatments due to variability between animals.

The controversial nature of ketogenic dietary patterns has led to the evaluation of their physiological impact through the use of different animal models as a surrogate for human subjects (Ellenbroek et al., 2014; Yang et al., 2021; Zhang et al., 2024). In the present study, carbohydrate energy was replaced with beef tallow as the main energy source in a nutritionally balanced swine diet formulated for maintenance under similar caloric and protein concentrations. A goal of the present study was to induce a state of dietary ketosis. Nutritional ketosis was not detected in the urine of the LCHF group during the third week and only 3 LCHF gilts had traces (5 mg/dL) of ketones in the fourth. That said, the stagnation of BW gain and the steady decline in FD over time and loss of visceral fat indicate a metabolic shift that can be attributed to the low‐carb, high‐fat diet independent of a measurable state of ketosis.

### 4.1 Animal performance and body composition

A proposed advantage of consuming a dietary pattern that results in physiological ketosis is associated with appetite suppression and sustained satiety (Gibson et al., 2015). In the present study, the LCHF gilts consumed less feed (evidenced by lower ADFI), despite being offered the same amount of feed on a per kg of BW basis as their pair‐fed CON counterpart. In other words, LCHF gilts appeared to reach satiety sooner and stopped eating. The LCHF group lost body fat, while maintaining a stable BW. Most notably, after 32 d on test, CON gilts had 1.74 times more visceral fat (internal belly fat) than their LCHF counterparts. Our results agree with human trials (Basolo et al., 2022; Cicero et al., 2015; Paoli et al., 2019) reporting a general conservation of muscle mass coinciding with body weight reduction attributed to body fat mass loss. Still others (Hall et al., 2016) have suggested that the effects of improved lean‐to‐fat ratio from adherence to a ketogenic diet can only be achieved after 12 weeks or more of dietary ketogenic compliance. Our observations suggest that individuals seeking to lose body fat could make noticeable body composition changes in a relatively short period of time.

Human subjects adhering to a ketogenic diet have reported a common side effect of constipation. Ketogenic and high fiber diets are both recommended as a nutrient management strategy for glycemia, but the combined effect may be superior whereby the low‐carb, high‐fat ketogenic diet modulates insulin secretions, increases fat oxidation, and increases satiety, while insoluble fiber improves gut health and blunts circulating glucose spikes by slowing glucose absorption and increases satiety associated with gut fill (Rossi et al., 2026). In the present study, NDF (cellulose, hemicellulose, and lignin) represented 44.31% of the diet on a dry matter basis, while ADF (cellulose and lignin) was 32.13%. Cellulose and lignin are non‐soluble nutrients and the ADF faction was used to calculate the non‐fiber carbohydrates used to determine kcals/kg of feed fed for the CON and LCHF diets. Further, a recent study by Mayer et al. (Mayer et al., 2026) found swine diets containing up to 40% concentration of soybean hulls resulted in increased fecal and urine nitrogen excretion, decreased apparent total tract digestibility, and decreased the metabolizable energy of the diet. Therefore, soy hulls added to the LCHF may have contributed to the early satiety (and lower feed consumed) of the LCHF gilts, or the increased concentration of soy hulls in the LCHF diet restricted the availability of digestible energy. Future research should consider the impact of fiber and/or high fat on the apparent total tract digestibility. That said, both diets were formulated to equally meet the dietary needs as determined by NRC guidelines for swine of this age, stage of reproduction, and sex. If there were restrictions in the digestibility of nutrients, the pigs would have lost weight. In the present study, the LCHF pigs did not stop growing; they stopped depositing fat. In other words, they lost fat mass but did not lose body weight. This outcome supports a positive impact of a LCHF dietary strategy for individuals seeking to lose body fat while maintaining lean body mass.

Our findings of a larger liver (expressed as a percentage of BW) in the LCHF versus CON gilts are in contrast with a meta‐analysis evaluation of the physiological impact of a ketogenic diet on liver health where, on average, ketogenic diets decreased metabolic risk factors for non‐alcoholic fatty liver disease, reduced hepatic concentrations of adipose, increased insulin sensitivity, and a reduced expression of liver enzymes (Emanuele et al., 2025). Despite the heavier liver present in the LCHF group, no difference in crude fat content was observed between CON and LCHF treatments and therefore no indication of early development of non‐alcoholic fatty liver disease.

### 4.2 Blood chemistry panel

Multiple authors have reported following a ketogenic diet produces a significant reduction of fasting glucose and insulin concentrations (indicating significant improvements in glycemia) of healthy and T2D subjects (Cicero et al., 2015; Domosławska‐Żylińska et al., 2024; Paoli et al., 2019; Romano et al., 2019; Wong et al., 2021; Yancy et al., 2005; Yang et al., 2021; Zhang et al., 2024). In the present study, fasting glucose levels were not different between dietary treatments. There was a diet by time tendency whereby blood glucose concentration of the LCHF group steadily increased over time compared to CON that remained flat for the first 14 d and then declined at d 32. A possible explanation for the diet × time tendency may be the initial development of a keto‐adaptation state whereby liver function physiologically adapts to protect glycogen reserves when only mild nutritional ketosis is induced. This physiological adaptation is likely due to the provision of adequate dietary protein to support gluconeogenesis, thus limiting the need to mobilize endogenous protein sources and thereby sparing muscle tissue wasting (Huang et al., 2020).

Dietary treatment did not influence blood pH in the present study. This is an important observation because uncontrolled diabetic ketoacidosis where ketonemia can exceed 20 mmol/L can lead to a systemic pH reduction and death. Additionally, sodium concentration was not affected by diet or by diet over time, but potassium was significantly higher in CON and ionic calcium tended to be greater in LCHF. The tendency for the LCHF gilts to possess higher circulating Ca concentration may be partially explained by the slightly higher amount of Ca in the LCHF diet (0.64 ± 0.03% of diet vs. 0.52 ± 0.01% in CON) and the short feeding period. Contrasting results regarding calcium content in blood have been reported for ketogenic diets (Buscemi et al., 2021; Kenig et al., 2019; Lee et al., 2022; Molteberg et al., 2021). That said, the present study indicates that consumption of a ketogenic diet for a 4‐week period did not disrupt blood chemistry homeostasis, with fasted blood parameters remaining at or below normal reference ranges (Lee et al., 2024).

### 4.3 Insulin and insulin receptor (IR)

Adherence to a ketogenic diet can result in restricted capacity for β‐cell expansion and proliferation, reduced β‐cell mass, and restricted pancreatic insulin expression resulting in a decrease of circulating insulin (Bielohuby et al., 2013; Ellenbroek et al., 2014; Her et al., 2020). In the present study, the percentage of active β‐cells and the percentage of the area filled by β‐cells within the pancreatic islets tended to be higher in the LCHF group, suggesting an enhancement of the pancreatic architecture and β‐cell function. This would suggest greater capacity to produce insulin for release into circulation. During ketogenesis, the sensitivity of the insulin receptor is promoted, improving the ability of insulin to regulate glucose uptake and metabolism (Cornier et al., 2005; Yuan et al., 2020). That said, this response is conditional on a reduction of serum insulin. We found the relative intensity of insulin receptor fluorescence used as a measure of the presence of insulin receptor specific proteins present on the surface of skeletal muscles was numerically higher in *longissimus* and was significantly increased in the *semimembranosus* of the LCHF group. The increase in insulin receptors on these muscle fibers may be contributing to a muscle sparing effect associated with the anabolic influence of insulin on myofibrillar protein synthesis (Barrea et al., 2024; Schenk et al., 2009). Extended periods of nutritional ketosis can generate metabolic adaptations that suppress insulin‐induced glucose uptake by skeletal muscle and improve maintenance of muscle glycogen (Wilson et al., 2020). Ketosis prevents muscle wasting by decreasing the gluconeogenic demand for glucogenic amino acids from muscle tissue due to the hepatic generation of ketone bodies that provide a fuel alternative to glucose (Basolo et al., 2022; Veech, 2004). Moreover, an adequate protein intake can contribute to maintaining constant insulin levels (insulinotropic effect) and promote secretion of growth hormone (GH) that preserves lean body mass and muscle (Barrea et al., 2024). Therefore, the greater expression of insulin receptors present in the *semimembranosus* of the present study supports anabolic regulation and maintenance of this powerful muscle of locomotion in response to consumption of a low‐carb, high‐fat diet.

## 5 CONCLUSION

Consumption of a nutritionally balanced low‐carbohydrate, high‐fat diet improved body composition, pancreatic insulin production mechanics, and *semimembranosus* insulin receptor density using swine as a biomedical model for human females. Ketogenic diets aim to reduce blood glucose. However, blood glucose concentrations in the current study tended to increase in the LCHF gilts over time. It is important to view these statistical tendencies with caution due to the small sample size and short feeding duration. These data provide preliminary justification to further pursue the metabolic impact of low‐carb, high‐fat or ketogenic diets on body composition and the physiological factors that impact overweight and obesity using swine as a model for humans.

## AUTHOR CONTRIBUTIONS

**Eric P. Berg:** Conceptualization; data curation; formal analysis; funding acquisition; investigation; methodology; project administration; resources; software; supervision; validation; visualization. **Natalie Acosta‐Castellanos:** Data curation; formal analysis; investigation; project administration. **Shelly Tiede:** Formal analysis; investigation; methodology; validation. **Christopher J. Byrd:** Conceptualization; data curation; formal analysis; funding acquisition; investigation; methodology; project administration; resources; supervision. **Pawel Borowicz:** Formal analysis; investigation; resources; software. **Jennifer M. Young:** Data curation; formal analysis; investigation; methodology; project administration; resources; supervision; validation; visualization.

## FUNDING INFORMATION

The project was partially funded by the North Dakota Agricultural Experiment station and by the North Dakota State University EXPLORE (Undergraduate Excellence in Research and Scholarly Activity) program.

## CONFLICT OF INTEREST STATEMENT

The authors declare no conflict of interest.

## ETHICS STATEMENT

All procedures were approved by the North Dakota State University (NDSU) Institutional Animal Care and Use Committee (Protocol #20220002).

## Data Availability

Data are available upon request.
